# High efficacy and tolerability of daratumumab-based regimens in first-line treatment of patients with immunoglobulin light-chain amyloidosis: a real-world evidence analysis

**DOI:** 10.3389/fimmu.2026.1960805

**Published:** 2026-10-28

**Authors:** Yajuan Cui, Yunya Luo, Ji Li, Lingli Yuan, Zhihua Wang, Hongling Peng, Ruijuan Li

**Affiliations:** Department of Hematology, The Second Xiangya Hospital, Central South University, Changsha, Hunan, China

**Keywords:** immunoglobulin light chain amyloidosis, AL amyloidosis, daratumumab, immunotherapy, efficacy, tolerability, outcomes

## Abstract

**Background:**

Daratumumab was the first monoclonal antibody targeting cluster of differentiation 38 (CD38) to demonstrate high response rates in both untreated and pretreated cases of immunoglobulin light-chain (AL) amyloidosis.

**Patients and methods:**

We conducted a single-center, retrospective real-world case series to evaluate the efficacy and safety of daratumumab as a first-line treatment option. Daratumumab was administered with bortezomib and dexamethasone (D-Vd), bortezomib, cyclophosphamide, and dexamethasone (D-VCd), or low-dose dexamethasone (Dd).

**Results:**

Forty-four patients were eligible, including 14 patients with stage IIIb. By the 6-month milestone (N = 36), 21 (59%) patients achieved at least complete response (CR), 10 (28%) patients achieved very good partial response (VGPR), and 5 (14%) patients achieved partial response (PR). The cardiac response rate was 50% at 3 months, 69% at 6 months, and 84% at 12 months. The renal response rate reached 40% by the 6-month mark. Importantly, hematologic, cardiac, and renal response rates were generally comparable between stage IIIb and non-stage IIIb patients. After a median follow-up of 13 (range 2-45) months, 6 (14%) patients died, all with stage IIIb disease. The median overall survival (OS) was not reached, with a 6-month survival rate of 93% and a 1-year survival rate of 84%. Univariate analysis showed that Mayo stage IIIb, difference between involved and uninvolved free light chains (dFLC) ≥ 300 mg/L, and a time to treatment of more than 12 months were associated with poor overall survival. Lymphocytopenia emerged as the most prevalent hematologic adverse effect, with grade 3-4 lymphocytopenia observed in 32% of patients. Among non-hematologic adverse reactions, infection was observed in 39% of patients.

**Conclusion:**

Daratumumab demonstrates significant efficacy in treating newly diagnosed AL amyloidosis, with excellent hematologic and organ response rates, a remarkable safety profile, and favorable tolerability among AL amyloidosis patients. However, early cardiac mortality before responses could manifest underscores that advanced cardiac stage remains an adverse prognostic factor for OS.

## Introduction

Immunoglobulin light-chain (AL) amyloidosis is a lethal disease characterized by amyloid deposition within vital organs, particularly the heart and kidneys, leading to end-organ dysfunction and death (1). The outcome of patients with AL amyloidosis strongly correlates with the severity of organ involvement, especially the heart (2). Despite the lack of therapies proven to disrupt fibril deposition, the mainstay of AL amyloidosis therapy involves anti–plasma cell therapy to halt or reduce light chain production. Cyclophosphamide, bortezomib, and dexamethasone (VCd) represented a standard treatment for patients prior to 2020 (3, 4). Other treatments, such as melphalan and dexamethasone, immunomodulatory drugs, or bendamustine, were used as alternatives (5). However, patients with Mayo stage IIIb continue to exhibit a poor prognosis, with a median overall survival (OS) of 4–6 months, highlighting the need for more effective treatment strategies (2, 4, 5).

The development of targeted anti‐clonal therapies, like daratumumab, has transformed the treatment landscape, leading to the accelerated approval of a daratumumab-based regimen by the Food and Drug Administration for this rare disease on January 15, 2021, based on the ANDROMEDA study. The study demonstrated a 53.3% hematologic complete response (CR) rate in the daratumumab plus VCd (D-VCd) group versus 18.1% in the VCd alone group, and major organ remission and progression-free survival (PFS) also favored the daratumumab group (6).

In Asians, the overall hematologic CR rate was 58.6% for D-VCd versus 9.7% for VCd, with six-month cardiac response rates of 46.7% versus 4.8%, respectively. It has been previously demonstrated that deep and rapid hematologic responses from daratumumab can improve outcomes for this population. However, not all the patients in the real world could tolerate the increased toxicity of these upfront 4-drug regimens, leaving the management of these patients as an unmet need, especially those considered too frail for clinical trials. We conducted this retrospective single-institution study to evaluate the clinical characteristics, treatment efficacy, and outcomes in patients with AL amyloidosis treated with daratumumab-based contemporary regimens.

## Patients and methods

### Patients and treatment

This retrospective study enrolled 44 consecutive patients with newly diagnosed AL amyloidosis from a single referral center in the Department of Hematology, The Second Xiangya Hospital, Central South University, from January 2021 to December 2024. Patients were excluded if they met the diagnostic criteria for multiple myeloma, monoclonal gammopathy of undetermined significance (MGUS), or polyneuropathy, organomegaly, endocrinopathy, monoclonal gammopathy, and skin changes (POEMS) syndrome. Bone marrow examination, including aspiration and biopsy, was performed in all patients to assess the percentage of clonal plasma cells. Patients had a histopathological diagnosis of AL amyloidosis. For histopathological confirmation of amyloidosis, the biopsy site was selected according to clinical presentation and suspected organ involvement. Biopsy of the involved organ was the preferred choice; if this was not feasible, skin biopsy could be performed, given its convenience and relatively high positive rate for amyloid deposition. If amyloid deposition was not detected on skin biopsy, bone marrow specimens were subjected to Congo red staining. In our cohort, amyloid deposition was confirmed by skin biopsy in 27 patients, renal biopsy in 12 patients, endomyocardial biopsy in 1 patient, and bone marrow biopsy in 4 patients. Organ involvement was defined according to the 2005 International Society of Amyloidosis (ISA) consensus criteria (7): (1) Kidney involvement: 24-h urine protein excretion ≥0.5 g/day, predominantly albuminuria; (2) Cardiac involvement: mean left ventricular wall thickness >12 mm on echocardiography in the absence of other cardiac causes, or N-terminal pro-B-type natriuretic peptide (NT-proBNP) >332 ng/L in the absence of atrial fibrillation or renal insufficiency; (3) Hepatic involvement: total liver span >15 cm in the absence of heart failure, or when alkaline phosphatase (ALP) > 1.5 times the institutional upper limit of normal. The Mayo 2004 criteria were used for patient stratification, with Mayo stage IIIb defined by both cTnT ≥0.035 ng/mL and NT‑proBNP ≥332 pg/mL, with NT‑proBNP ≥8500 pg/mL. (8). Clinical data were prospectively registered in a database, and all patients were treated with intravenous daratumumab. The choice of combined treatment was based on renal and cardiac function, additional comorbidities, and performance status. This study was approved by the institutional review board of The Second Xiangya Hospital. All patients provided their written informed consent for data collection and analysis, in accordance with the Declaration of Helsinki.

### Response assessment

Hematologic response and organ responses were assessed using the current consensus criteria for AL amyloidosis (9, 10). Hematologic response was stratified into stringent complete response (sCR; CR with involved free light chain (iFLC) ≤ 20 mg/L and difference between involved and uninvolved free light chains (dFLC) ≤ 10 mg/L), complete response (CR, normal free light chain (FLC) ratio and negative serum and urine immunofixation), very good partial response (VGPR, dFLC < 40 mg/L), partial response (PR, dFLC decrease > 50%), and stable disease (SD). Organ response was stratified into CR (nadir NT-proBNP ≤ 400 pg/mL; nadir proteinuria ≤ 200 mg per 24 h; a 50% decrease in abnormal alkaline phosphatase value and/or a radiographic reduction in liver size of at least 2 cm); VGPR (>60% reduction in a parameter not meeting the complete organ response definition); PR (31%-60% reduction in a parameter); and no response (NR; ≤30% reduction in an organ response parameter). The overall response rate (ORR) includes CR, VGPR, and PR.

### Statistical analysis

Descriptive statistics are reported as medians with ranges, and frequencies are reported as percentages. Time to event was calculated from the date of initiation of therapy until the date of death by any cause, the date of disease progression or recurrence, or the date of last follow‐up. Survival curves were plotted with the Kaplan‐Meier method, and comparisons were made using the log‐rank test. P values < 0.05 were considered statistically significant. Analyses were performed using SPSS (IBM SPSS Statistics, version 26, IBM Corp., Armonk, NY, USA).

## Results

### Baseline characteristics

Patients’ baseline demographic and clinical characteristics are shown in **Table 1**. Forty-four patients were enrolled, with a median age of 59 (35-77) years and a male-to-female ratio of 1.3:1. Forty patients had cardiac involvement (91%), 30 (68%) had renal involvement, and 3 (7%) had liver involvement. The median level of NT-proBNP was 3276.0 ng/L (range 26.9->35000.0 ng/L), the median 24-hour urinary albumin was 2.155 g (0.000-16.193 g), the median ALP was 79 U/L (35–434 U/L), and the median dFLC was 300.0 mg/L (15.9-16094.8 mg/L). Seventeen (39%) patients had an impaired estimated glomerular filtration rate (eGFR) < 60 mL/min. Four patients (9%) were stratified as Mayo 2004 stage I, 13 (30%) as stage II, 13 (30%) as stage IIIa, and the remaining 14 (32%) as stage IIIb (**Table 1**). All patients received primary treatment with daratumumab: 21 (48%) in combination with bortezomib and dexamethasone (D-Vd), 17 (39%) in combination with bortezomib, cyclophosphamide, and dexamethasone (D-VCd), and 6 (14%) in combination with low-dose (8 mg/week or less) dexamethasone (Dd) (**Table 1**). For patients with stage IIIb, eight (57%) received D-Vd, 3 patients (21%) received D-VCd, and 3 patients (21%) received Dd.

**Table 1:** Baseline characteristics of patients with immunoglobulin light-chain amyloidosis.

Characteristic	Total (n = 44)
Age, y	59 (35-77)
≥65 y, %	13(30)
Male, %	25(57)
Organ involvement, %	
Heart	40(91)
Renal	30(68)
Liver	3(7)
NT-proBNP, ng/L^#^	3276 (26.9->35000)
24-hour urinary albumin, g	2.155 (0.000-14.593)
Renal function status	
eGFR < 60 mL/min	17(39)
ALP, U/L	79(35-434)
dFLC, mg/L	300.0(15.9-16094.8)
Mayo 2004 stratification	
I	4(9)
II	13(30)
IIIa	13(30)
IIIb	14(32)
Involved free light chain	
Kappa, %	8(18)
Lambda, %	36(82)
Primary treatment	
D-Vd, %	21(48)
D-VCd, %	17(39)
Dd, %	6(14)

NT-proBNP, N-terminal pro-B-type natriuretic peptide; eGFR, estimated glomerular filtration rate; ALP, alkaline phosphatase; dFLC, difference between involved and uninvolved free light chains; D-Vd, daratumumab in combination with bortezomib and dexamethasone; D-VCd, daratumumab with bortezomib, cyclophosphamide, and dexamethasone; Dd, daratumumab with low-dose (8 mg/week or less) dexamethasone.

^#^The upper reportable limit of the NT-pro-BNP assay was 35,000 pg/mL.

### Hematologic and organ responses

The median number of courses of initial daratumumab-based treatment was 5 (1-13). At the 1-month landmark (N = 44), 11 (25%) patients achieved ≥CR, 16 (36%) patients achieved VGPR, 13 (30%) patients achieved PR, and 4 (9%) did not achieve a hematologic response (stable disease, SD). At the 3‐month landmark (N = 42), 21 (50%) achieved ≥CR, 12 (29%) patients achieved VGPR, 7 (17%) patients achieved PR, and 2 (5%) patients had SD. At the 6‐month landmark (N = 36), 21 (59%) patients achieved ≥CR, 10 (28%) patients achieved VGPR, and 5 (14%) patients achieved PR. At the 12‐month landmark (N = 22), 14 (64%) patients achieved ≥CR and 8 (36%) patients achieved VGPR (**Figure 1A**). Hematologic response (PR or better) was achieved by 91% of patients within 1 month, 95% within 3 months, and 100% within 6 months, respectively. Sixty-one percent of the patients achieved ≥VGPR within 1 month, 78% within 3 months, 86% within 6 months, and 100% within 12 months, respectively (**Figure 1A**). The 3-month cardiac response rate was 50%, the 6‐month rate was 69%, and the 12‐month rate was 84% (**Figures 1B, C**). The renal response rate was 36% and 50% at 6 and 12 months, respectively (**Figures 1B, D**). No liver response was achieved.

**Figure 1:**
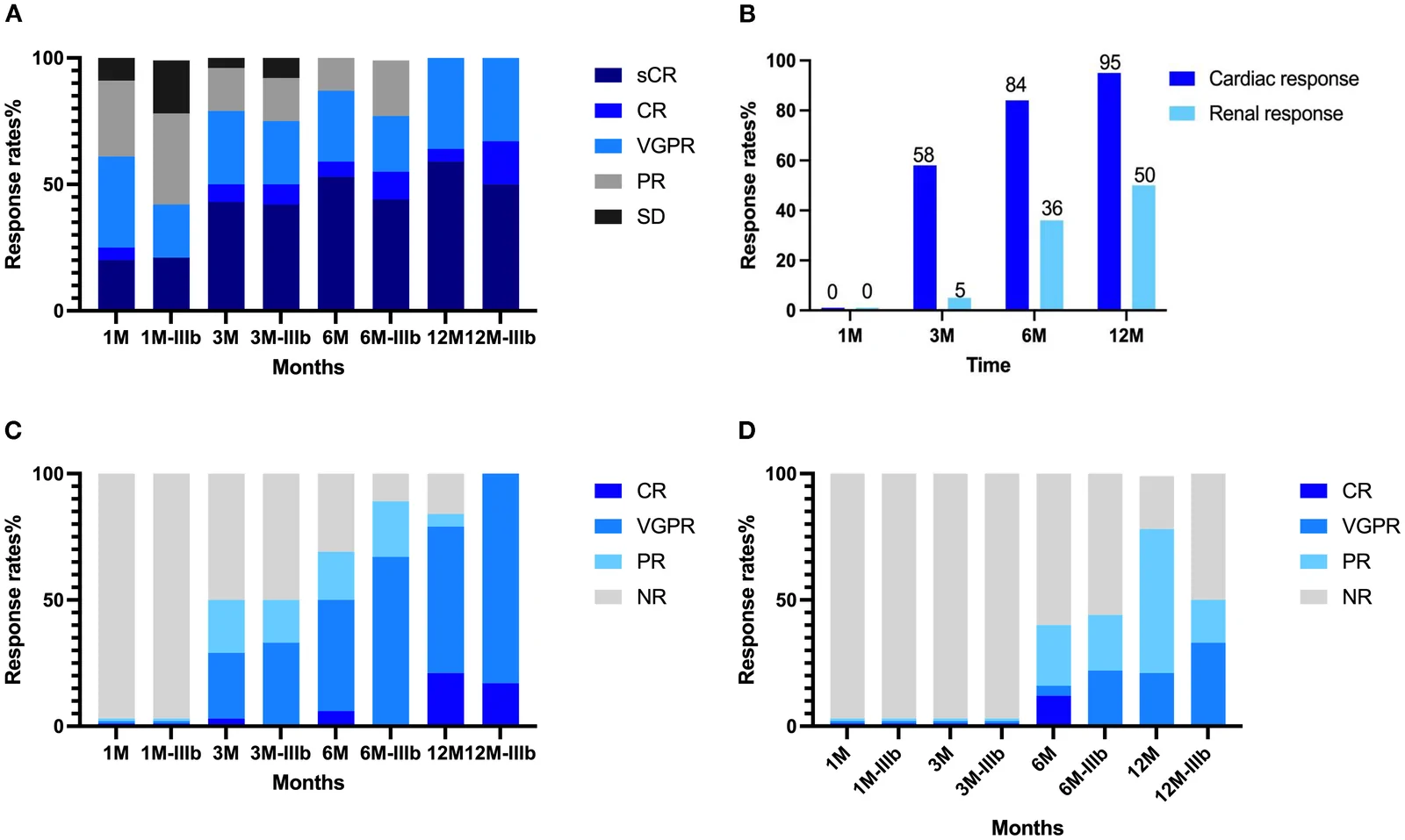
**(A)** Hematologic response rates in patients treated with daratumumab-based regimens. sCR, stringent complete response; CR, complete response; VGPR, very good partial response; PR, partial response; SD, stable disease. **(B)** Organ response rates in patients treated with daratumumab-based regimens. **(C)** Cardiac response rates in patients treated with daratumumab-based regimens. **(D)** Renal response rates in patients treated with daratumumab-based regimens.

For the 14 stage IIIb patients, at the 1‐month landmark (N = 14), 3 (21%) achieved ≥CR, 3 (21%) achieved VGPR, 5 (36%) achieved PR, and 3 (21%) did not achieve a hematologic response (SD). At the 3-month landmark (N = 12), 6 (50%) achieved ≥CR, 3 (25%) achieved VGPR, 2 (17%) achieved PR, and 1 (8%) had NR. At the 6-month landmark (N = 9), 5 (55%) achieved ≥CR, 2 (22%) achieved VGPR, and 2 (22%) achieved PR. At the 12-month landmark (N = 6), 4 (67%) achieved ≥CR and 2 (33%) achieved VGPR (**Figure 1A**). The cardiac response rate was 50% (6/12), 89% (7/9), and 100% (6/6) at the 3-, 6-, and 12-month landmarks, respectively (**Figure 1C**). The renal response rate was 44% and 50% at the 6- and 12‐month landmarks, respectively (**Figure 1D**).

### Safety

Grade 3–4 hematologic adverse reactions included 7 cases (16%) of neutropenia, 14 cases (32%) of lymphocytopenia, and 4 cases (9%) of anemia. Non-hematologic adverse reactions included 6 cases (14%) of infusion-related reactions, all of which were grade 1-2. There were a total of 26 cases (59%) of infection, with 9 cases of grade 1-2, 16 cases of grade 3, and 1 case of grade 5, including 22 cases of respiratory infections and 4 cases of gastrointestinal infections.

### Survival

The median follow-up for the whole cohort was 12 (2-45) months. Six patients (14%) died, all with stage III disease. The median overall survival (OS) was not reached, with a 6-month survival rate of 90%, a 1-year survival rate of 84%, and a 2-year survival rate of 84% (**Figure 2**). In the univariate analysis, Mayo stages I, II, and IIIa were associated with better median OS than Mayo stage IIIb (p = 0.0002) (**Figure 3A**). A baseline dFLC level of less than 300 mg/L was correlated with superior OS compared to dFLC ≥ 300 mg/L (p = 0.0111) (**Figure 3B**). A time to treatment of less than 12 months was correlated with superior OS compared to a time to treatment of more than 12 months (p = 0.0064) (**Figure 3C**). Achieving at least VGPR at 1 month was associated with superior OS compared to not reaching this benchmark (p = 0.0489) (**Figure 3D**).

**Figure 2:**
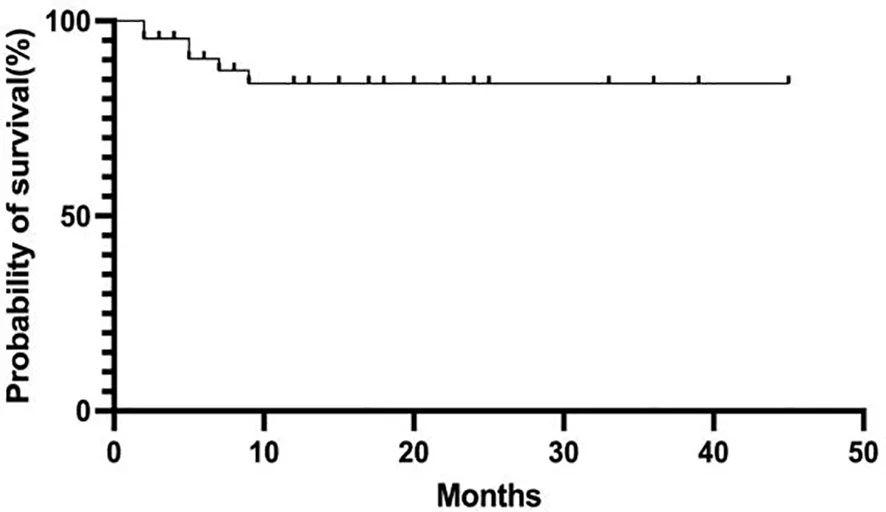
Overall survival of the total cohort of patients with AL amyloidosis.

**Figure 3:**
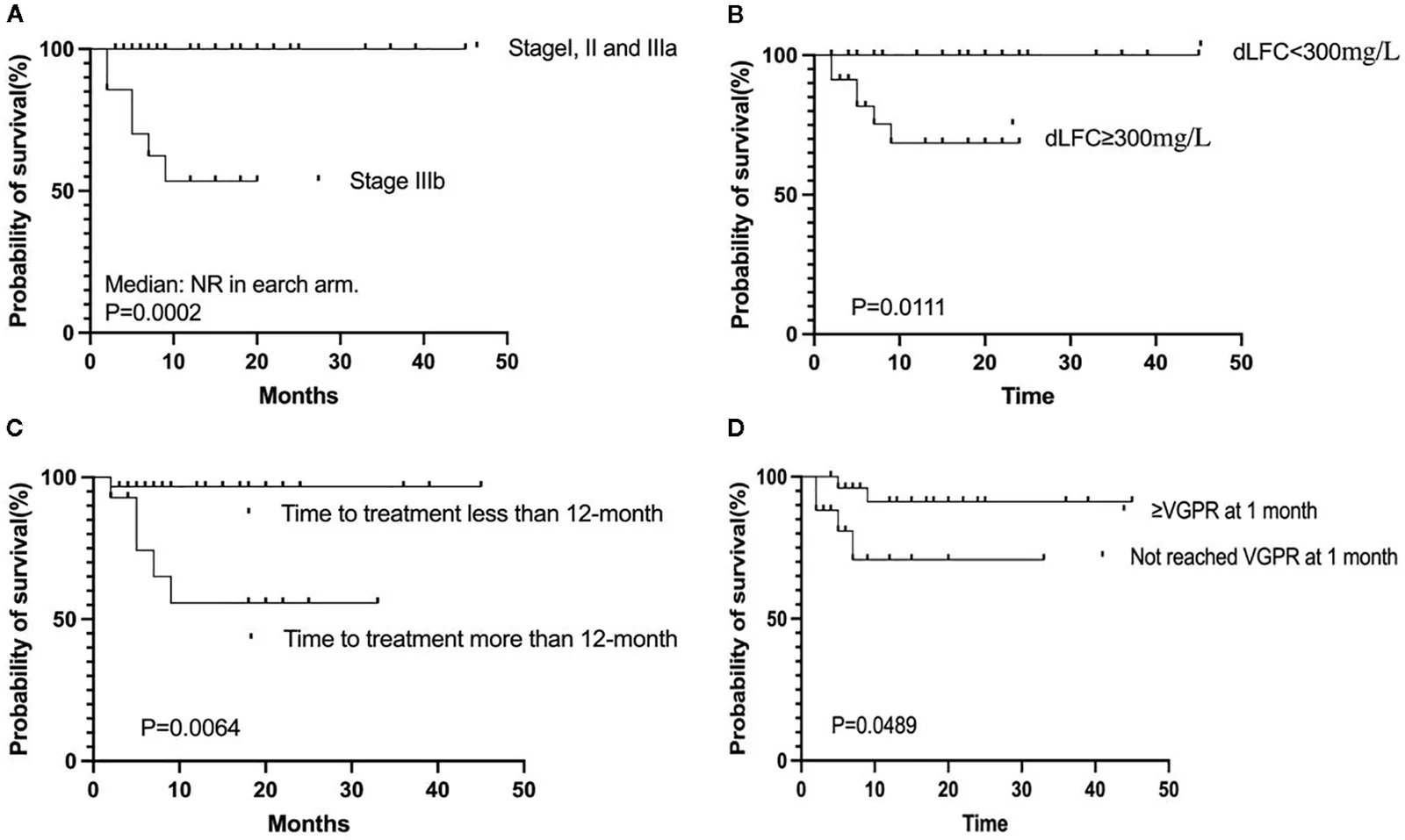
Overall survival stratified by Mayo stage **(A)**, baseline dFLC level **(B)**, time to treatment **(C)**, and achievement of at least a hematologic very good partial response at 1 month **(D)**. dFLC, difference between involved and uninvolved free light chains.

## Discussion

Daratumumab is a monoclonal antibody targeting cluster of differentiation 38 (CD38), with direct on-tumor (11) and immunomodulatory mechanisms of action (12, 13). It can induce a deep and rapid hematologic response in both newly diagnosed and previously treated patients with cardiac AL amyloidosis. Early death and treatment intolerance are more likely to occur in patients with stage IIIb disease, so the regimens included in this study were individualized and included D-Vd, D-VCd, and Dd. In this retrospective analysis, the complete hematologic and cardiac response rates in our series were 59% and 69%, respectively, at 6 months after treatment initiation, consistent with those in the D-VCd group from the ANDROMEDA population (14) and in real-world studies of daratumumab (15, 16).

D-VCd had a higher hematologic CR rate and deeper and more rapid hematologic and organ responses versus VCd (6, 14). As daratumumab and bortezomib have shown potent activities toward AL amyloidosis, the benefit and toxicity of an alkylating agent in this combination warrant further evaluation. Yumeng Zhang et al (17) reported that the addition of cyclophosphamide to bortezomib and dexamethasone (Vd) was not associated with improved hematologic response, best organ response, or overall survival in a retrospective study involving 144 patients with AL amyloidosis. In our series, only 17 of 44 patients received cyclophosphamide, and the response rate in the D-VCd population was consistent with that of the total cohort, indicating whether cyclophosphamide is still necessary in the daratumumab and bortezomib era. More clinical trials and real-world research are needed to determine the best combination of regimens for hematologic and organ responses.

The rapid, deep hematologic responses observed with daratumumab support its use as a novel standard of care for initial treatment of patients with AL amyloidosis. In our cohort, patients treated with daratumumab achieved their first hematologic response at about 1.3 months, in accordance with the median time to response of about 1-1.4 months in pretreated and untreated patients with AL amyloidosis (15, 18). Cardiac response rates were 50.9% and 33.3% in the stage II and IIIa subgroups, respectively, of the D-VCd group, compared with 29.6% and 15.9%, respectively, in the corresponding VCd subgroups, and the incidence of all reported adverse events (AEs) was lower with D-VCd than with VCd, supporting the use of D-VCd in patients with cardiac involvement (19). In our study, 50% of the patients achieved early cardiac remission, with only two early deaths observed within 3 months. The use of daratumumab has, to some extent, improved the survival of these patients. We found that, at the corresponding time points, stage IIIb and non-stage IIIb patients achieved generally comparable hematologic, cardiac, and renal response rates, suggesting that disease stage may have limited influence on treatment response in this cohort. Notably, the number of daratumumab treatment cycles appeared to be associated with organ response rates. This suggests that using daratumumab in patients with amyloidosis, including those with stage IIIb disease, can rapidly achieve hematologic and cardiac remission.

It is reported that the 6-month renal response rates were 57.1% in the D-VCd group and 37.5% in the VCd group among Asian patients, with no statistical difference between the groups (14). The renal response rate was 36% at 6 months and 50% at 12 months of follow-up. Renal response always occurs later than hematologic and cardiac responses, no patients achieved renal response at 3 months in our group. The renal response rates were 44% and 79% at 6 and 12 months, respectively, in the overall population. Among patients with stage IIIb disease, the renal response rates were 44% and 50% at 6 and 12 months, respectively. The renal response rates were in accordance with the analysis of ANDROMEDA (14), suggesting that daratumumab based regimens in first-line can yield a high renal response rate. Overall, the proportion of patients achieving at least VGPR in the renal response is lower than that for cardiac responses, the underlying mechanisms warrant further investigation. Living donor kidney transplantation provided remarkable advantages for recipients with AL amyloidosis (20, 21). The sustained use of proteasome inhibitors and anti-CD38 monoclonal antibodies, with higher rates of complete hematologic remission and longer response durations, has been described as minimizing the risk of relapse with preserved kidney function in patients who received living donor kidney transplantation (22).

In our research, no deaths occurred among the 30 non-stage IIIb patients during the follow-up period. In contrast, six deaths (6/14) were observed in the stage IIIb group before an adequate response could be achieved, one due to severe infection, and five due to cardiac events. For patients with cardiac involvement, the incidence of cardiac events was numerically greater with D-VCd than with VCd, but the rate of grade 3 or 4 events was similar. The exposure-adjusted incidence rate for cardiac events was lower with D-VCd than with VCd (19). Daratumumab has shown an acceptable safety profile and encouraging efficacy in patients with newly diagnosed AL amyloidosis (6, 14) and in those with relapsed/refractory AL amyloidosis (23). In our study, there were few severe AEs associated with treatment. Early deaths within 3 months were observed in 2 patients stratified as stage IIIb. Early use of daratumumab could reduce dFLC and improve cardiac remission, but early mortality still occurred. Even in the era of daratumumab, we should pay more attention to patients with stage IIIb disease due to the dismal prognosis across different cohorts, with lower response rates and poor survival.

Patients with severe cardiac involvement at baseline have not experienced the same level of benefit as the overall population of patients with AL amyloidosis, especially patients with stage IIIb disease. In our series, patients stratified as stage IIIb had worse survival. It is critical to provide the best possible care with the most effective treatment regimens as early as possible, to halt organ damage and improve prognosis (24, 25). In the Mayo 2012 staging system (27), dFLC ≥ 180 mg/L is considered an adverse prognostic factor; elevated dFLC has also been considered a poor prognostic factor in other studies. Patients with dFLC < 50 mg/L presented with less frequent and less severe cardiac involvement, but higher renal involvement (26). In our group, patients had more frequent and more severe cardiac involvement, with a median dFLC of 300 mg/L. Daratumumab could reduce dFLC within a median time of 7 days. In our series, dFLC ≥ 180 mg/L was not an adverse prognostic factor, while dFLC ≥ 300 mg/L was an adverse prognostic factor, partially because of the use of daratumumab and the limited number of patients.

### Study limitations

A limitation of this retrospective, single-center case series is the small number of patients, particularly in the stage IIIb subgroup (n = 14). Although these attributes are likely reflective of the higher disease burden in patients in higher cardiac stages, they may also have independently affected the outcomes, including older age, worse Eastern Cooperative Oncology Group performance status, COVID 19 infectious, and the relatively heterogeneous study population. Our study lacks detailed information on the nature of arrhythmias during the cardiac events. Longer follow-up is needed to assess late cardiac and renal responses and determine whether these findings are associated with differences in overall survival.

## Conclusions

Here, we report on daratumumab as an effective first-line treatment strategy for AL amyloidosis in a retrospective case series of 44 patients in a real-life setting. All patients rapidly achieved hematologic responses that deepened over time. A substantial proportion also showed cardiac and renal responses. The treatment was generally well tolerated, with no severe side effects from either daratumumab itself or any of the combination partners that were added over the course of the treatment. Focusing on the high-risk stage IIIb subgroup, we observed hematologic and organ response rates comparable to those of non-stage IIIb patients. However, mortality in stage IIIb patients was concentrated early in the treatment course, predominantly from cardiac events occurring before responses could be achieved, underscoring that advanced cardiac stage remains an adverse prognostic factor for overall survival despite effective anti-clonal therapy. Given the retrospective design and the small stage IIIb sample size, these findings should be considered hypothesis-generating and warrant validation in larger, ideally prospective studies. Daratumumab-based therapy is a promising index treatment strategy for AL amyloidosis, including stage IIIb disease, but early cardiac mortality remains a critical unmet need in this subgroup.

## Data Availability

The original contributions presented in the study are included in the article/supplementary material. Further inquiries can be directed to the corresponding author.

## References

[1] Merlini G Bellotti V . Molecular mechanisms of amyloidosis. N Engl J Med. (2003) 349:583–96. doi: 10.1056/nejmra023144 12904524

[2] Wechalekar AD Schonland SO Kastritis E Gillmore JD Dimopoulos MA Lane T . A European collaborative study of treatment outcomes in 346 patients with cardiac stage III AL amyloidosis. Blood. (2013) 121:3420–7. doi: 10.1182/blood-2012-12-473066 23479568

[3] Mikhael JR Schuster SR Jimenez-Zepeda VH Bello N Spong J Reeder CB . Cyclophosphamide-bortezomib-dexamethasone (CyBorD) produces rapid and complete hematologic response in patients with AL amyloidosis. Blood. (2012) 119:4391–4. doi: 10.1182/blood-2011-11-390930 22331188 PMC3557400

[4] Palladini G Sachchithanantham S Milani P Gillmore J Foli A Lachmann H . A European collaborative study of cyclophosphamide, bortezomib, and dexamethasone in upfront treatment of systemic AL amyloidosis. Blood. (2015) 126:612–5. doi: 10.1182/blood-2015-01-620302 25987656

[5] Palladini G Schonland S Merlini G Milani P Jaccard A Bridoux F . The management of light chain (AL) amyloidosis in Europe: clinical characteristics, treatment patterns, and efficacy outcomes between 2004 and 2018. Blood Cancer J. (2023) 13:19. doi: 10.1038/s41408-023-00789-8 36697388 PMC9876983

[6] Kastritis E Palladini G Minnema MC Wechalekar AD Jaccard A Lee HC . Daratumumab-based treatment for immunoglobulin light-chain amyloidosis. N Engl J Med. (2021) 385:46–58. doi: 10.1056/nejmoa2028631 34192431

[7] Gertz MA Comenzo R Falk RH Fermand JP Hazenberg BP Hawkins PN . Definition of organ involvement and treatment response in immunoglobulin light chain amyloidosis (AL): a consensus opinion from the 10th International Symposium on Amyloid and Amyloidosis, Tours, France, 18-22 April 2004. Am J Hematol. (2005) 79:319–28. doi: 10.1002/ajh.20381 16044444

[8] Dispenzieri A Gertz MA Kyle RA Lacy MQ Burritt MF Therneau TM . Serum cardiac troponins and N-terminal pro-brain natriuretic peptide: a staging system for primary systemic amyloidosis. J Clin Oncol. (2004) 22:3751–7. doi: 10.1201/9781420037494-24 15365071

[9] Palladini G Dispenzieri A Gertz MA Kumar S Wechalekar A Hawkins PN . New criteria for response to treatment in immunoglobulin light chain amyloidosis based on free light chain measurement and cardiac biomarkers: impact on survival outcomes. J Clin Oncol. (2012) 30:4541–9. doi: 10.1200/jco.2011.37.7614 23091105

[10] Muchtar E Dispenzieri A Wisniowski B Palladini G Milani P Merlini G . Graded cardiac response criteria for patients with systemic light chain amyloidosis. J Clin Oncol. (2023) 41:1393–403. doi: 10.1200/jco.22.00643 36215675 PMC10489422

[11] Overdijk MB Verploegen S Bogels M van Egmond M Lammerts van Bueren JJ Mutis T . Antibody-mediated phagocytosis contributes to the anti-tumor activity of the therapeutic antibody daratumumab in lymphoma and multiple myeloma. MAbs. (2015) 7:311–21. doi: 10.1080/19420862.2015.1007813 25760767 PMC4622648

[12] Krejcik J Casneuf T Nijhof IS Verbist B Bald J Plesner T . Daratumumab depletes CD38+ immune regulatory cells, promotes T-cell expansion, and skews T-cell repertoire in multiple myeloma. Blood. (2016) 128:384–94. doi: 10.1182/blood-2015-12-687749 27222480 PMC4957162

[13] Casneuf T Adams HC van de Donk N Abraham Y Bald J Vanhoof G . Deep immune profiling of patients treated with lenalidomide and dexamethasone with or without daratumumab. Leukemia. (2021) 35(2):573–84. doi: 10.1038/s41375-020-0855-4 PMC7862054 32457357

[14] Suzuki K Wechalekar AD Kim K Shimazaki C Kim JS Ikezoe T . Daratumumab plus bortezomib, cyclophosphamide, and dexamethasone in Asian patients with newly diagnosed AL amyloidosis: subgroup analysis of ANDROMEDA. Ann Hematol. (2023) 102:863–76. doi: 10.1007/s00277-023-05090-z 36862168 PMC9998577

[15] Jeryczynski G Antlanger M Duca F Binder-Rodriguez C Reiter T Simonitsch-Klupp I . First-line daratumumab shows high efficacy and tolerability even in advanced AL amyloidosis: the real-world experience. ESMO Open. (2021) 6:100065. doi: 10.1016/j.esmoop.2021.100065 33667762 PMC7937667

[16] Shen KN Miao HL Gao YJ Cao XX Zhou DB Su W . [Efficacy and safety of daratumumab in the treatment of advanced light chain amyloidosis]. Zhonghua Xue Ye Xue Za Zhi. (2022) 43(1):31–34. doi: 10.3760/cma.j.issn.0253-2727.2022.01.007 PMC8980654 35231990

[17] Zhang Y Duncanson L Brayer J Reu F Hansen D Alsina M . Adding cyclophosphamide to bortezomib and dexamethasone was not associated with improved outcomes of patients with newly diagnosed light chain amyloidosis: A retrospective study. Clin Lymphoma Myeloma Leuk. (2022) 22:e770–6. doi: 10.1016/j.clml.2022.04.003 35504808 PMC10766144

[18] Kaufman GP Schrier SL Lafayette RA Arai S Witteles RM Liedtke M . Daratumumab yields rapid and deep hematologic responses in patients with heavily pretreated AL amyloidosis. Blood. (2017) 130:900–2. doi: 10.1182/blood-2017-01-763599 28615223

[19] Minnema MC Dispenzieri A Merlini G Comenzo RL Kastritis E Wechalekar AD . Outcomes by cardiac stage in patients with newly diagnosed AL amyloidosis: phase 3 ANDROMEDA trial. JACC CardioOncol. (2022) 4:474–87. doi: 10.1016/j.jaccao.2022.08.011 36444227 PMC9700253

[20] Angel-Korman A Stern L Sarosiek S Sloan JM Doros G Sanchorawala V . Long-term outcome of kidney transplantation in AL amyloidosis. Kidney Int. (2019) 95:405–11. doi: 10.1016/j.kint.2018.09.021 30580886

[21] Sathick IJ Rosenbaum CA Gutgarts V Landau H . Kidney transplantation in AL amyloidosis: is it time to maximize access?. Br J Haematol. (2020) 188(3):e1–e4. doi: 10.1111/bjh.16246 31730739

[22] Guillen E Montagud-Marrahi E Rodriguez DM Hermida E Padilla RA Blasco M . Living donor kidney transplantation is an effective option of renal replacement therapy in patients with light-chain amyloidosis (AL). Ann Hematol. (2020) 99:2961–2. doi: 10.1007/s00277-020-04230-z 32862282

[23] Sanchorawala V Sarosiek S Schulman A Mistark M Migre ME Cruz R . Safety, tolerability, and response rates of daratumumab in relapsed AL amyloidosis: results of a phase 2 study. Blood. (2020) 135(18):1541–47. doi: 10.1182/blood.2019004436 PMC7193185 31978210

[24] Al Hamed R Bazarbachi AH Bazarbachi A Malard F Harousseau JL Mohty M . Comprehensive Review of AL amyloidosis: some practical recommendations. Blood Cancer J. (2021) 11:97. doi: 10.1038/s41408-021-00486-4 34006856 PMC8130794

[25] Palladini G Milani P Malavasi F Merlini G . Daratumumab in the Treatment of Light-Chain (AL) Amyloidosis. Cells. (2021) 10(3):545. doi: 10.3390/cells10030545 PMC7998921 33806310

[26] Qiu Y Zhang CL Shen KN Su W Feng J Zhang L . Clinical presentation and prognosis of light-chain amyloidosis patients with unmeasurable free light-chain levels. Ann Hematol. (2018) 97:2465–70. doi: 10.1007/s00277-018-3460-0 30056579

[27] Kumar S Dispenzieri A Lacy MQ Hayman SR Buadi FK Colby C . Revised prognostic staging system for light chain amyloidosis incorporating cardiac biomarkers and serum free light chain measurements. J Clin Oncol. (2012) 30(9):989–95. doi: 10.1200/JCO.2011.38.5724 PMC3675680 22331953

